# Prevalence of mood, panic and eating disorders in obese patients referred to bariatric surgery: patterns of comorbidity and relationship with body mass index

**DOI:** 10.1007/s40519-021-01236-y

**Published:** 2021-06-16

**Authors:** Margherita Barbuti, Giulio E. Brancati, Alba Calderone, Paola Fierabracci, Guido Salvetti, Francesco Weiss, Giulia Carignani, Ferruccio Santini, Giulio Perugi

**Affiliations:** 1 Psychiatry 2 Unit, Department of Clinical and Experimental Medicine, University Hospital of Pisa, Via Savi 10, 56126 Pisa, Italia; 2 Endocrinology Unit, Department of Clinical and Experimental Medicine, Obesity and Lipodystrophy Research Center, University Hospital of Pisa, Pisa, Italy

**Keywords:** Mood disorders, Panic disorder, Eating disorders, Obesity, Bariatric surgery

## Abstract

**Purpose:**

We aimed at investigating the lifetime prevalence of mood, eating and panic disorders in a large sample of obese patients referred to bariatric surgery. We also explored the patterns of psychiatric comorbidity and their relationship with Body Mass Index (BMI).

**Methods:**

The sample was composed of patients consecutively referred for pre-surgical evaluation to the Obesity Center of Pisa University Hospital between January 2004 and November 2016. Clinical charts were retrieved and examined to obtain sociodemographic information, anthropometric variables and lifetime psychiatric diagnoses according to DSM-IV criteria.

**Results:**

A total of 871 patients were included in the study; 72% were females, and most patients had BMI ≥ 40 kg/m^2^ (81%). Overall, 55% of the patients were diagnosed with at least one lifetime psychiatric disorder. Binge eating disorder (27.6%), major depressive disorder (16%), bipolar disorder type 2 (15.5%), and panic disorder (16%) were the most common psychiatric diagnoses. Mood disorders showed associations with panic disorder (OR = 2.75, 95% CI = 1.90–3.99, χ^2^ = 41.85, *p* = 0.000) and eating disorders (OR = 2.17, 95% CI 1.64–2.88, χ^2^ = 55.54, *p* = 0.000). BMI was lower in patients with major depressive disorder (44.9 ± 7.89) than in subjects without mood disorders (46.75 ± 7.99, *p*_adj_ = 0.017).

**Conclusion:**

Bariatric patients show high rates of psychiatric disorders, especially binge eating and mood disorders. Longitudinal studies are needed to explore the possible influence of such comorbidities on the long-term outcome after bariatric surgery.

**Level of evidence:**

V, cross sectional descriptive study.

## Introduction

Obesity, defined by a body mass index (BMI = kg/m^2^) of 30 or greater, is a chronic disease with a multifactorial etiopathogenesis, involving genetics, environment, metabolism, lifestyle and behavioral components. In recent decades, obesity has reached pandemic proportions, especially in Western countries. Excess weight is one of the most common risk factors underlying major chronic diseases such as heart disease, stroke, cancer, chronic respiratory disease and diabetes, which are the leading cause of mortality worldwide [1].

At present, bariatric surgery is the most durable weight-loss treatment for severe obesity. It is recommended in patients with a BMI greater than 40 kg/m^2^, or in those with a lower BMI if obesity-related comorbidities, such as type 2 diabetes and cardiovascular diseases, are present. Surgical treatment for obesity results in greater weight loss than conservative treatments, such as lifestyle interventions and pharmacological therapies, and is associated with significant improvement in obesity-related comorbidities and reduced mortality rate [2].

Psychiatric assessment is widely recommended during the multidisciplinary evaluation performed prior to bariatric surgery. In fact, psychiatric disorders are thought to have an impact on various post-surgical outcomes, both in the short- and the long-term, including weight loss, mental health and quality of life [3, 4]. However, at present, most authors believe that psychiatric comorbidity does not contraindicate surgery, with a few exceptions such as current substance abuse [5].

Although no conclusive data have been produced, there is strong evidence of a close bidirectional relationship between obesity and various psychiatric disorders [5–7]. This association appears to strengthen with increasing BMI and to be moderated by several variables, such as age, gender, or socioeconomic status [8, 9]. In addition, bariatric patients seem to exhibit significantly higher rates of psychopathology than obese individuals who do not seek treatment or obese individuals who adhere to conservative programs that emphasize dietary restriction or promote weight control [4, 10].

Binge eating, mood and anxiety disorders are the most often detected conditions in obese bariatric patients [11]. Although binge-eating disorder (BED) is not limited to obese individuals, it is most common in this group and is often associated with various forms of psychopathology, including mood and anxiety symptoms [12]. Among mood disorders, depression is the most investigated condition in obese individuals seeking weight-loss treatment, whereas bipolar disorder is often overlooked. The latter is a chronic recurrent disorder characterized by abnormal fluctuations in mood state and energy. Bipolar type I and II disorders differ from unipolar depressive disorder since patients also exhibit pronounced episodes of mood elevation (manic and hypomanic episodes, respectively) [13]. Finally, among anxiety disorders, most previous reports on bariatric patients have focused on panic disorder. This psychiatric condition is characterized by recurrent panic attacks, which are sudden, intense bursts of anxiety or fear accompanied by a range of physical symptoms and maladaptive behaviors [14].

There are few comprehensive studies investigating psychiatric comorbidity through structured interviews in large populations of pre-surgical obese patients. Therefore, the aim of the present research was to provide lifetime prevalence rates of mood, eating, and panic disorders in a very large Italian sample of bariatric patients. A secondary objective was to explore patterns of psychiatric comorbidity and their relationship with BMI.

## Patients and methods

### Patient sample and data collection

The sample of this cross sectional and observational study was composed of adult (≥ 18 years) obese bariatric patients. Without any exclusion criteria, we included in the study sample all subjects consecutively referred for bariatric surgery to the Obesity Center of the Endocrinology Unit in Pisa University Hospital, between January 2004 and November 2016. All patients were routinely interviewed by licensed psychiatrist during the pre-surgical evaluation.

Clinical charts were retrieved and examined. For any given patient, two independent researchers retrieved the data and filled a pre-defined data abstraction form. Any disagreement was resolved by consensus after discussion. All the medical records available were searched for sociodemographic information, anthropometric variables, i.e., weight and BMI, and lifetime diagnoses of mood, anxiety and eating disorders. Particularly, the diagnoses of major depressive disorder, bipolar disorders, panic disorder, BED, bulimia and anorexia nervosa were registered. To better define psychiatric diagnoses, the Structured Clinical Interview for DSM-IV Axis I disorders (SCID-I) [15] was used during the clinical interviews conducted over the years by trained psychiatrists.

### Statistical analysis

Descriptive statistics were used to summarize the characteristics of the sample. The prevalence of mood, panic and eating disorders were computed and confidence intervals with 95% confidence level (95% CI) were obtained according to Clopper and Pearson [16]. Pearson’s chi-squared tests were used to identify: (1) pairwise associations between mood disorders, panic disorder and eating disorders; (2) associations between major depressive vs. bipolar disorders and panic or eating disorders, within patients affected by mood disorders; (3) differences in gender proportions among all psychiatric comorbidity groups. A post-hoc analysis based on residuals was applied to the latter test, using the Benjamini–Hochberg method to adjust the significance level for multiple comparisons. Analyses of Variance (ANOVA) were conducted to assess significant differences of age and BMI between psychiatric comorbidity groups, the latter analysis including effects of age and gender. A Tukey’s post-hoc test was used whenever the ANOVA led to a statistically significant result to retrieve significant comparisons between variables. A statistical significance level of *p* < 0.05 was set for all tests. All the analyses were performed using R Statistical Software (Foundation for Statistical Computing, Vienna, Austria).

## Results

Our sample was composed of 871 obese patients referred for bariatric surgery. The female gender accounted for 72% of the sample and age ranged between 18 and 69 years, with a mean of approximately 45 years (Table 1). Weight ranged between a minimum of 76 kg to a maximum of 264 kg, with a mean of 126.48 ± 26.07 kg. BMI ranged between 31 kg/m^2^ and 87 kg/m^2^, with a mean of 46.32 ± 7.85 kg/m^2^. As expected, the majority of patients was diagnosed with class III obesity (BMI ≥ 40 kg/m^2^), with more than three fourths of patients affected (*N* = 707, 81.2%). A lower number of patients was diagnosed with class II obesity (35 kg/m^2^ ≤ BMI < 40 kg/m^2^) (*N* = 139, 16.0%), while an almost negligible group of subjects was affected by class I obesity (BMI < 35 kg/m^2^) (*N* = 25, 2.8%). Marital status was available for 679 patients, educational level for 670 patients, and employment status for 780 subjects. More than half of the patients were married/cohabiting (69%) and employed (65%) at the time of the evaluation; the vast majority of the sample had a middle or high school diploma (respectively, 41% and 39%).

**Table 1:** Sociodemographic characteristics of obese patients referred to bariatric surgery (*N* = 871)

Female gender (*n*, %)	629 (72.2)
Age, years (mean, SD)	45.36 (10.84)
BMI, kg/m^2^ (mean, SD)	46.32 (7.85)
Marital status (*n*, %)	
Never married	131 (19.3)
Married	470 (69.2)
Divorced	62 (9.1)
Widowed	16 (2.4)
Education level (*n*, %)	
Degree	68 (10.2)
High school diploma	262 (39.1)
Middle school diploma	273 (40.8)
Primary school diploma	67 (10.0)
Employment status	
Employed	509 (65.3)
Unemployed	192 (24.6)
Retired	57 (7.3)
Student	22 (2.8)

*BMI* body mass index

Mood and eating disorders were highly prevalent in the sample (Table 2). Lifetime major depressive disorders or bipolar disorders were diagnosed in 338 patients (38.7%); BED, bulimia nervosa and anorexia nervosa were diagnosed in 259 subjects (29.7%). BED was the most prevalent condition, involving most of the patients with eating disorders (93.1%). One of three subjects with anorexia nervosa and more than a half of patients with bulimia nervosa (*N* = 19, 54.3%) were indeed also diagnosed with BED. Among mood disorders, major depressive disorder and bipolar disorder type 2 were the most frequently diagnosed, followed, in order, by cyclothymia/other specified bipolar disorder and bipolar disorder type 1. However, bipolar disorders represented the second most frequent diagnosis overall (*N* = 198, 22.7%, 95% CI = 20.0–25.7), exceeding both major depressive disorder and panic disorder. The latter two conditions showed the same prevalence.

**Table 2:** Lifetime prevalence of mood, panic and eating disorders in obese patients referred to bariatric surgery (*N* = 871)

	*N*	%	95% CI
Any psychiatric disorder	480	55.1%	51.7–58.5
Any affective disorder	338	38.7	35.5–42.0
Major depressive disorder	140	16.0	13.7–18.6
Bipolar disorder type 1	21	2.4	1.5–3.7
Bipolar disorder type 2	135	15.5	13.1–18
Cyclothymia/other specified bipolar disorder	42	4.8	3.5–6.4
Any eating disorder	259	29.7%	26.7–32.9
Binge-eating disorder	241	27.6	24.7–30.7
Bulimia nervosa	35	4.0	2.8–5.5
Anorexia nervosa	3	0.3	0.1–1
Panic disorder	140	16.0	13.7–18.6

**Fig. 1:**
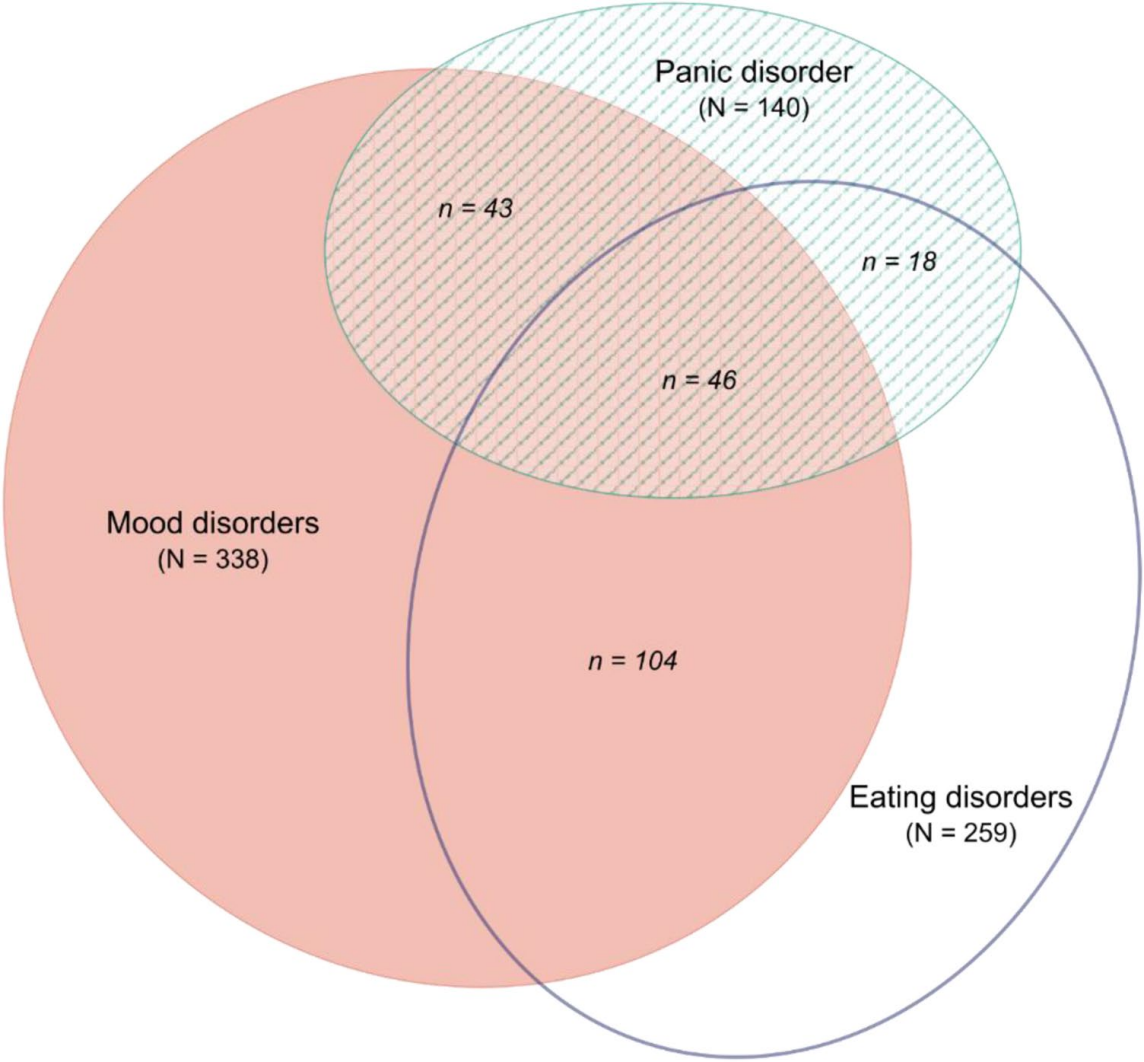
Patterns of comorbidity between mood, panic and eating disorders in obese patients referred to bariatric surgery (*N* = 871). “*N*” is used for three group sample size, while “*n*” indicates subgroup sample size

Significant pairwise associations between mood, panic and eating disorders were found in the whole sample (*N* = 871) (Fig. 1). Mood disorders showed the strongest association with panic disorder (OR 2.75, 95% CI 1.90–3.99, χ^2^ = 41.85, *p* = 0.000). In addition, twice as many patients were diagnosed with eating disorders among mood disordered subjects with respect to unaffected individuals (OR = 2.17, 95% CI = 1.64–2.88, χ^2^ = 55.54, *p* = 0.000). Also eating disorders and panic disorder were significantly associated (OR = 1.99, 95% CI = 1.38–2.86, χ^2^ = 19.49, *p* = 0.000), with most of comorbid patients also showing a mood comorbidity (46 of 64, 71.9%.

Within mood disordered patients (*N* = 338), no significant association between bipolar disorders and eating disorders was observed, with 56 of major depressive patients (40.0%) and 94 of bipolar patients (47.5%) being comorbid (χ^2^ = 1.57, *p* = 0.211). Conversely, a significant association between major depressive disorder and panic disorder comorbidity was found within mood disordered patients (*N* = 338, χ^2^ = 4.69, *p* = 0.030). Indeed, 46 of major depressive patients (32.9%) were diagnosed with panic disorder, compared to 43 of patients with bipolar disorder (21.7%). Multimorbid patients with mood, panic and eating disorders (*N* = 46), were equally represented in both the groups with major depressive disorder (*N* = 23, 16.4%) and bipolar disorders (*N* = 23, 11.6%) (*N* = 338, χ^2^ = 1.23, *p* = 0.267).

Gender proportions were found to significantly differ between diagnostic groups (χ^2^ = 43.78, df = 11, *p* = 0.000). Males were overrepresented in subjects without psychiatric comorbidity (143 of 391, 36.6%) as compared to all the other patients diagnosed with at least one among mood, panic and eating disorders (99 of 480 overall, 20.6%), as suggested by post-hoc residuals analysis (residual = 5.23, *p*_adj_ = 0.000). As for age, a significant effect of mood disorders [F (2, 859) = 6.14, *p* = 0.002] and a significant interaction between the effects of mood disorders and eating disorders [F (2, 859) = 3.40, *p* = 0.034] on age emerged. Post-hoc contrasts revealed that patients with major depressive disorder (*N* = 140, age = 47.63 ± 10.79) were significantly older than patients without mood disorders (*N* = 533, age = 44.95 ± 10.98, *p*_adj_ = 0.002). In addition, patients with major depressive disorder without eating disorders (*N* = 84, age = 50.61 ± 10.24) were significantly older than patients without both the disorders (*N* = 424, age = 45.36 ± 11.05, *p*_adj_ = 0.001), with eating disorders only (*N* = 109, age = 43.35 ± 10.57, *p*_adj_ = 0.000), with major depressive disorder and eating disorders (*N* = 56, age = 43.16 ± 10.11, *p*_adj_ = 0.001), and with bipolar and eating disorders (*N* = 94, age = 42.5 ± 9.92, *p*_adj_ = 0.000). Finally, patients with bipolar and eating disorders were significantly younger than patients with bipolar disorders only (*N* = 104, age = 46.99 ± 10.28, *p*_adj_ = 0.035).

Only a significant effect of mood disorders on BMI was observed [F (2, 825) = 4.29, *p* = 0.014]. Post-hoc contrasts revealed that patients with major depressive disorder (*n* = 140, BMI = 44.9 ± 7.89) showed, on average, a significantly lower BMI than patients without mood disorders (*n* = 533, BMI = 46.75 ± 7.99, *p*_adj_ = 0.017).

## Discussion

This is, to our knowledge, the largest study (*n* = 871) exploring the prevalence of psychiatric disorders through the administration of a structured interview in a population of obese patients seeking for weight-loss surgical treatment. In particular, we investigated the prevalence of mood, panic and eating disorders, according to DSM-IV criteria. The psychiatric assessment was performed by trained psychiatrists and took place during the multidisciplinary pre-surgical evaluation of the patients.

Similarly to most of the previous reports [10, 17–25], almost three-quarters of the sample were women, the mean age was 45 years, and the vast majority of patients belonged to class III obesity. Overall, the sample showed a lower level of education than the general population in Italy [26]. In accordance with the dramatic increase over the years of the most severe forms of obesity [27], in the current sample, we found a higher rate of class III obesity (80%) than in a previous sample of bariatric patients recruited at the University Hospital of Pisa (66%) [28].

Consistently with the growing evidence of a particularly high rate of psychiatric comorbidities in obese patients referred to bariatric surgery, in the current sample, 55% of participants were diagnosed with at least one lifetime psychiatric disorders. Men showed a lower rates of any lifetime psychiatric disorder than women, in agreement with some previous reports [18, 24, 28]. However, gender differences may reflect a male tendency to minimize psychiatric conditions and to report them less frequently to the physician [29].

To date, there is still little clarity on the exact prevalence of psychiatric disorders in obese patients and, in particular, in bariatric subjects. The few studies addressing this issue often present small sample sizes and use heterogeneous methodologies, such as different interviewing strategies (e.g., in-person versus telephone-interview) and psychometric questionnaires. In addition, accurate estimates are difficult to obtain because of the tendency of bariatric patients to underreport psychiatric conditions to access surgery [4]. As a consequence, a wide range of lifetime psychiatric disorders rates were reported by different studies (37–81%), both in USA and in Europe [10, 19, 28, 30–32].

In our sample, the most frequent lifetime psychiatric conditions were mood disorders, affecting approximately 39% of the sample. Specifically, 16% of patients were diagnosed with major depressive disorder and 23% with bipolar spectrum disorders, among which bipolar disorder type II was the most represented (15.5%). In agreement with previous reports [17, 20, 25], patients with mood disorders showed higher rates of comorbidity with eating and panic disorders than other patients. Unexpectedly, panic disorder was found to be significantly associated with major depression but not bipolar disorder.

In previous studies assessing psychiatric comorbidity in bariatric subjects, the prevalence of major depressive disorder and bipolar disorder type II were more prone to vary, while bipolar disorder type I showed an almost stable prevalence of 1–5% [24]. Considering the entire spectrum of bipolar disorders, some authors have reported very low rates (1–6%) [10, 20, 21, 28] while others have found higher percentages (33–37%) [19, 25, 31]. In an Italian sample, Alciati et al. [33] observed an impressive prevalence of broad bipolar spectrum disorders (89%), according to Angst’s classification [34]. Finally, only four previous studies have found comparable or higher rates of bipolar spectrum disorders than depressive disorders in samples of bariatric subjects [19, 25, 31, 33]. The extreme variability in the prevalence of mood disorders probably stems from different operational criteria being used in previous studies. In addition, bipolar disorder type II is frequently underdiagnosed or misdiagnosed with major depressive disorder, due to the difficult retrospective assessment of hypomanic episodes [35]. Importantly, in two large samples of patients with a major depressive episode, the presence of obesity was associated with higher rates of bipolar spectrum disorders and (hypo)manic symptoms, suggesting that obesity could be considered a marker of bipolarity in major depressive patients [36, 37]. Consistently with our study, bipolarity features were observed in approximately one half of depressed obese patients [36].

High variability also exists in literature regarding the percentage of bariatric patients showing a comorbidity with BED (5–49%) [10, 18, 19, 21, 28, 30, 31] and panic disorder (1–30%) [10, 19, 28, 32]. In the current report, BED was detected in almost one third of the entire sample and represented the most frequent individual diagnosis. Obese patients with comorbid BED have been previously shown to exhibit significantly higher rates psychiatric disorders compared to obese patients without BED, in particular among bariatric surgery candidates [18, 23, 24, 28]. Overall, recent evidence suggests that the presence of an eating disorder may be a marker for other psychiatric conditions, primarily mood and anxiety disorders [38]. A common diathesis of emotional dysregulation and impulsivity, predisposing to compulsive and addictive behaviors, was proposed as a mediating mechanism between BED, obesity, and bipolar disorders [25].

Finally, in our sample, BMI was found to be lower in patients with major depressive disorder compared to subjects without mood disorders. In a previous study, Kalarchian et al. [21] have found higher BMI in subjects with comorbid psychiatric disorders compared to other patients, whereas Segura-Garcia et al. [17] found a lower BMI in subjects with bipolar spectrum disorder compared to the others. However, most studies have failed to find clear associations between BMI and particular psychiatric conditions [20, 23, 25, 28] and more homogeneous future studies addressing this issue are certainly needed.

## Conclusions

Given the increasing prevalence of severe obesity and the rise of bariatric surgery worldwide, the evaluation of psychiatric disorders in bariatric candidates appears to play an increasingly important role. While not representing absolute contraindications to surgery, various psychiatric conditions could impact the long-term outcome after the intervention, both in terms of weight loss and quality of life. Identifying and treating mental disorders could improve patients' behaviors before and after surgery with important clinical and therapeutic implications. An integrated model of care should be used to ensure individualized treatments aligned with patient needs and symptom severity. Psychological interventions (primarily, cognitive and behavioral therapy) and psychopharmacological treatment have demonstrated to impact on disordered/maladaptive eating behaviors, psychological distress, and quality of life and should be considered in patients who are at risk for weight regain and adverse psychosocial outcomes [39].

### Strengths and limitations

In our opinion, the major limitation of this study is that we were only able to explore a small group of psychiatric disorders because of the absence of a systematic evaluation of other diagnostic categories, such as neurodevelopmental disorders, that only recently received sufficient attention from researchers and clinicians. In addition, the psychiatric evaluation in this study was part of the pre-surgical assessment, which may have led patients to underestimate some aspects of their psychiatric status or history to access surgery. Further studies are needed in which the assessment process is strictly independent of the surgery approval process to better detect the presence of an underlying psychopathology. Another limitation is the absence of a control group of overweight patients, which would have been useful to unravel other differences in BMI among groups of patients. Finally, we were only able to provide data regarding lifetime psychiatric comorbidities, without indicating the prevalence of current disorders or the percentages of patients on psychopharmacological therapy at the time of assessment. On the other hand, the major strengths of our study are certainly the very large sample and the use of a structured diagnostic interview by trained psychiatrists to confirm psychiatric diagnoses.

### What is already known on this subject?

Variably high lifetime prevalence rates of several mental disorders have been reported in relatively small samples of obese bariatric patients.

### What this study adds?

This is, to our knowledge, the largest study confirming the high prevalence of psychiatric disorders, particularly mood disorders and binge eating disorder, in patients seeking bariatric surgery.

## Data Availability

Data are available on reasonable request from the corresponding author.
